# Triple infection with *Cryptococcus*, varicella-zoster virus, and *Mycobacterium abscessus* in a patient with anti-interferon-gamma autoantibodies: a case report

**DOI:** 10.1186/s12879-020-4949-4

**Published:** 2020-03-19

**Authors:** Pongprueth Rujirachun, Jirath Sangwongwanich, Methee Chayakulkeeree

**Affiliations:** 1 Department of Microbiology, Faculty of Medicine Siriraj Hospital, Mahidol University, Bangkok, Thailand; 2 Faculty of Medicine Siriraj Hospital, Mahidol University, Bangkok, Thailand; 3 Division of Infectious Diseases and Tropical Medicine, Department of Medicine, Faculty of Medicine Siriraj Hospital, Mahidol University, 2 Wanglang Road, Bangkoknoi, Bangkok, 10700 Thailand

**Keywords:** Anti-interferon-gamma autoantibody, Cryptococcus, Varicella-zoster virus, Nontuberculous mycobacteria, *Mycobacterium abscessus*

## Abstract

**Background:**

The most common infection in patients positive for anti-interferon-gamma autoantibodies (anti-IFN-γ AAbs) is disseminated nontuberculous mycobacterial (dNTM) infection. Here, we report a rare case of triple infection caused by *Cryptococcus*, varicella-zoster virus (VZV), and nontuberculous mycobacterium in a patient with anti-IFN-γ AAbs.

**Case presentation:**

A 53-year-old Thai man presented with a progressively enlarging right cervical mass with low-grade fever and significant weight loss for 4 months. He also developed a lesion at his left index finger. A biopsy of that lesion showed granulomatous inflammation with yeast-like organisms morphologically consistent with cryptococcosis. Serum cryptococcal antigen was positive. Histopathology of a right cervical lymph node revealed chronic granulomatous lymphadenitis, and the lymph node culture grew *Mycobacterium abscessus.* One month later, he complained of vision loss in his left eye and subsequently developed a group of painful vesicles at the right popliteal area of S1 dermatome. Lumbar puncture was performed and his cerebrospinal fluid was positive for VZV DNA. His blood test for anti-HIV antibody was negative. Anti-IFN-γ AAbs was positive, but test for anti-granulocyte-macrophage colony-stimulating factor autoantibodies (anti-GM-CSF AAbs) was negative. He was treated with amphotericin B plus fluconazole for cryptococcosis; a combination of amikacin, imipenem, azithromycin, and levofloxacin for dNTM infection; and, intravenous acyclovir for disseminated VZV infection. After treatment, our patient’s fever and cervical lymphadenopathy were subsided, and his vision and visual acuity were both improved.

**Conclusions:**

This is the first case of triple infection with cryptococcosis, VZV, and dNTM in a patient who tested positive for anti-IFN-γ AAbs and negative for anti-GM-CSF AAbs. This case will increase awareness and heighten suspicion of these infections in patients with the described presentations and clinical characteristics, and this will accelerate diagnosis and treatment.

## Background

Adult-onset immunodeficiency with anti-interferon-gamma autoantibodies (anti-IFN-γ AAbs) is a newly recognized immunodeficiency syndrome that is predominantly reported in Southeast Asians. The most common infection in patients with this condition is disseminated nontuberculous mycobacterial (dNTM) infection [1]. Other opportunistic infections, such as varicella-zoster virus (VZV) infection or cryptococcosis, may occur in patients with anti-IFN-γ AAbs as unusual sequential or concomitant infections [2]. However, simultaneous multiple infections in patients with anti-IFN-γ AAbs are very rare. In this report, we present a patient positive for anti-IFN-γ AAbs, but negative for anti-granulocyte-macrophage colony-stimulating factor autoantibodies (anti-GM-CSF AAbs) who had triple infection with *Cryptococcus*, VZV, and *Mycobacterial abscessus*.

## Case presentation

A 53-year-old man presented to a private hospital in March 2018 with a progressively enlarging right cervical mass, low-grade fever, and significant weight loss for 4 months. Physical examination revealed right cervical lymphadenopathy. Needle aspiration of an enlarged lymph node was performed and the findings were suggestive of lymphadenitis without evidence of malignancy.

**Fig. 1:**
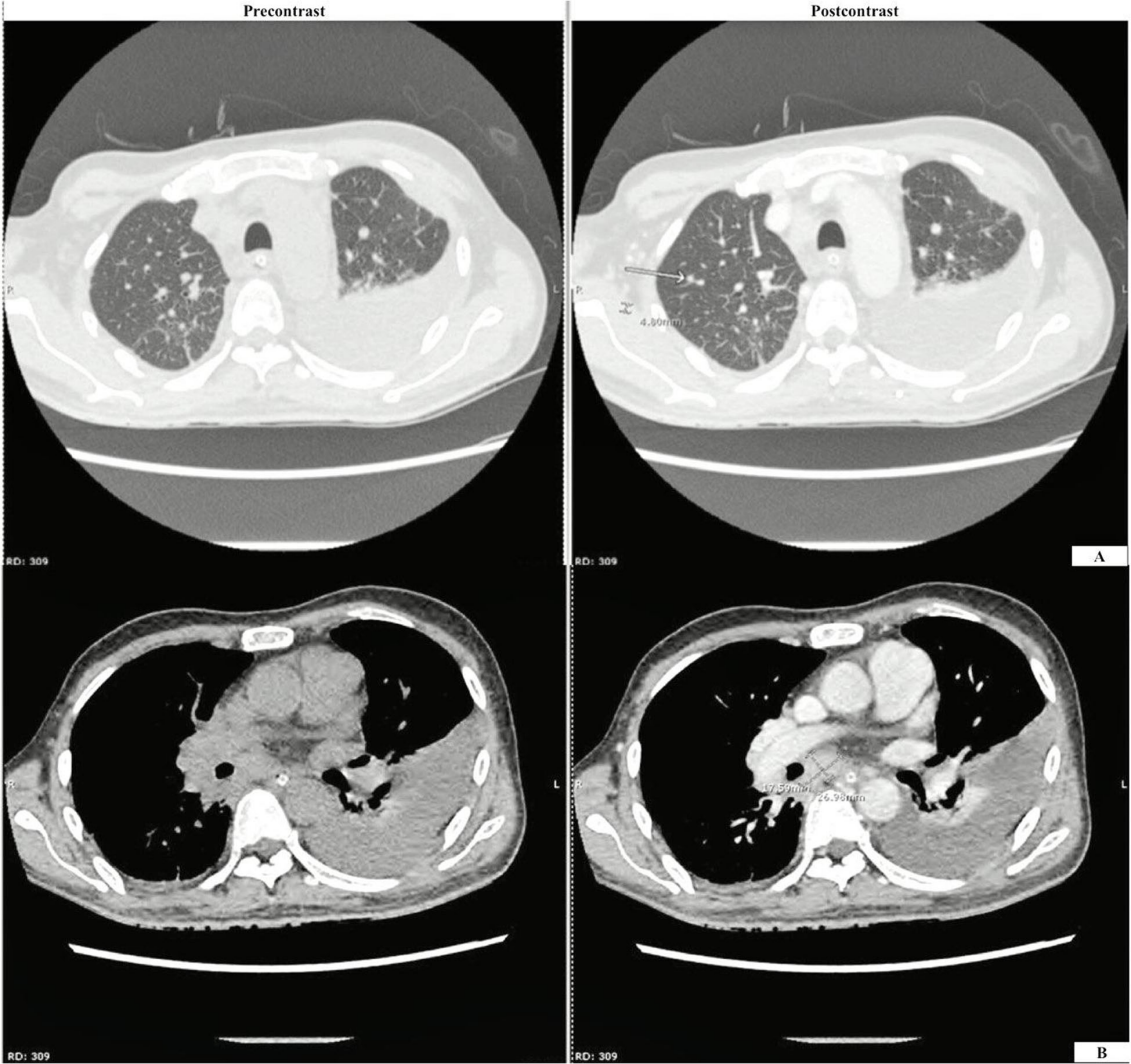
CT chest with contrast showed a 4.5 mm non-calcified nodule at the RUL (**a**) and multiple mediastinal mass lymphadenopathies measuring up to 2.6 × 1.8 cm at the subcarinal region (**b**)

**Fig. 2:**
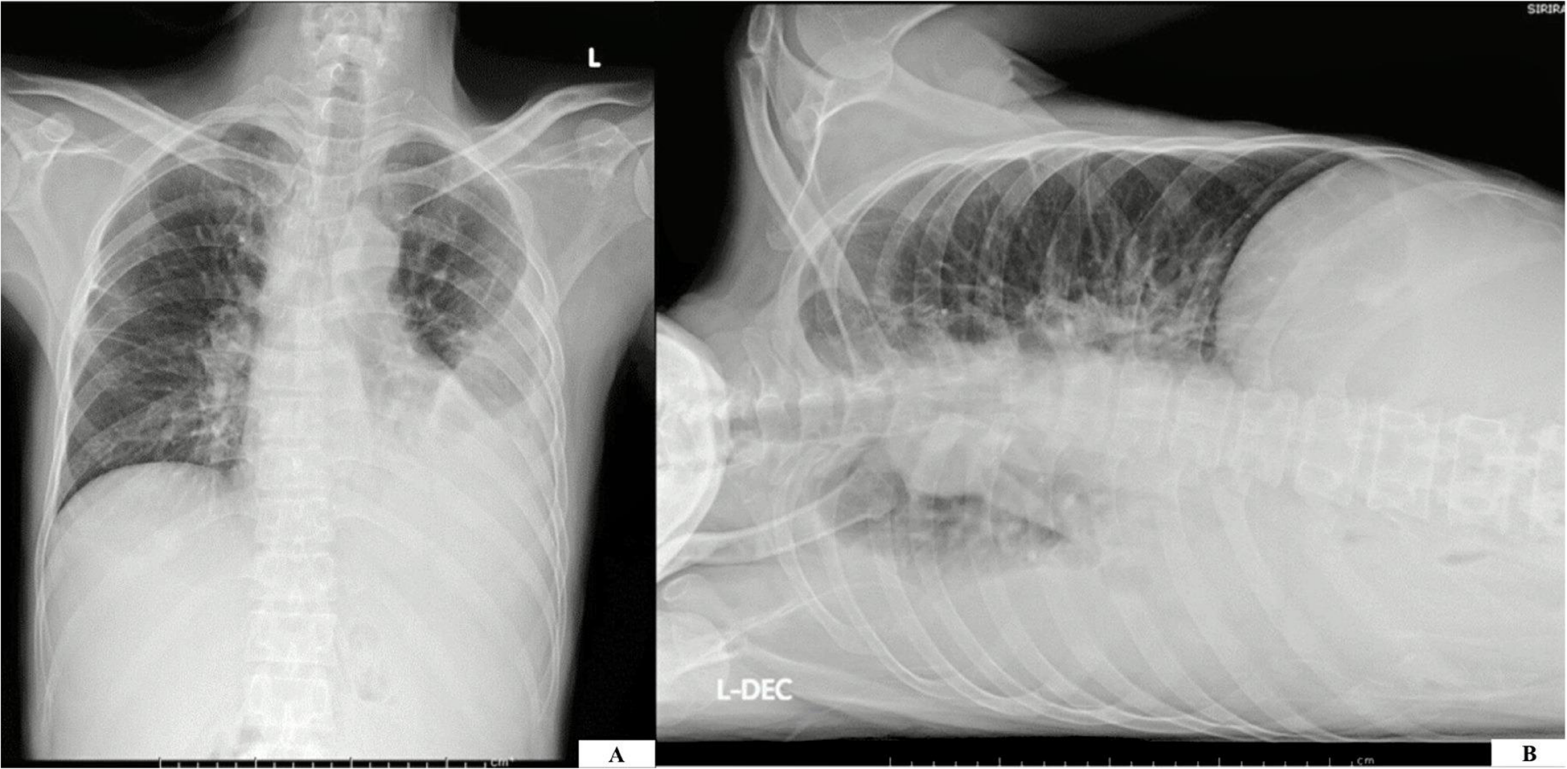
Chest radiography with the patient in the PA upright (**a**) and left lateral decubitus (**b**) positions revealed massive left pleural effusion

Four months later, he returned to the same hospital with progressive fatigue. Physical examination showed mild anemia, and a chronic ulcer at his left index finger was noted. Complete blood count revealed hemoglobin 9.0 g/dL, hematocrit 28.2%, white blood cell (WBC) count 9.9 × 10^9/^L (74.1% neutrophils, 14.6% lymphocytes, 3.5% monocytes, 6.9% eosinophils, and 0.9% basophils), and a platelet count of 207 × 10^9^/L. Blood test for anti-HIV antibody was negative. Right paratracheal mass was suspected from the chest radiograph. Chest computerized tomography showed a non-calcified nodule (4.5 mm in diameter) at the right upper lung with enlargement of multiple mediastinal lymph nodes (Fig. 1). Biopsy of a right cervical lymph node revealed chronic granulomatous lymphadenitis. Tuberculous lymphadenitis was suspected and empirical anti-tuberculosis therapy was commenced with rifampicin 600 mg/day, isoniazid 300 mg/day, pyrazinamide 1.6 g/day, and ethambutol 1.1 g/day. During admission, he developed dyspnea and his follow-up chest radiograph revealed left pleural effusion (Fig. 2). Thoracentesis was performed and the pleural fluid profile revealed a total cell count of 2.00 × 10^11^/L, RBC count of 0.00 × 10^11^/L, WBC count of 2.00 × 10^9^/L (25% neutrophils, 70% lymphocytes, 2% mesothelial cells, and 3% macrophages), adenosine deaminase (ADA) 7.21 IU/L, and cytology for malignancy was negative. The patient was then referred to our center for further investigation and treatment. The Faculty of Medicine Siriraj Hospital, Mahidol University is a 2300-bed national tertiary referral hospital that is located in Bangkok, Thailand.

Upon arrival from the referring hospital, our patient’s histopathological slides were reviewed. The biopsied tissue from his left index finger showed cryptococcosis with granulomatous reaction, and the biopsy from the right cervical lymph node showed chronic granulomatous inflammation with multiple minute foci and a small number of yeasts. Although fungal cultures from the left index finger, a right cervical lymph node, and blood were negative, serum cryptococcal antigen was positive by latex agglutination test (IMMY, Norman, OK, USA) with a titre of 1:32. Anti-IFN-γ AAbs was sent and the result showed positive. Antibodies to IFN-γ in serum were measured by in-house enzyme-linked immunosorbent assay (ELISA). The average optical density (OD) from two independent tests was greater than or equal to one, which is considered positive. The cut-off OD was determined using in-house data that was based on a comparison between a group of patients with this syndrome and more than 100 healthy controls. Anti-GM-CSF AAbs was also sent, but that result was negative. Amphotericin B 0.7 mg/kg/day and fluconazole 800 mg/day were given as induction therapy for 4 weeks, followed by oral fluconazole 800 mg as consolidation treatment. After antifungal treatment, the lymphadenopathy was not improved. Accordingly, a repeat biopsy of a right cervical lymph node was performed to identify another causative organism. The culture of that biopsied lymph node revealed *Mycobacterium abscessus*. The patient was diagnosed as interferon-gamma autoantibodies with disseminated cryptococcosis and *Mycobacterium abscessus* infection. He was treated with a combination of amikacin 750 mg/day, imipenem 3 g/day, azithromycin 500 mg/day, and levofloxacin 750 mg/day. After antimicrobial treatment for 1 month, our patient’s fever and cervical lymphadenopathy were subsided.

**Fig. 3:**
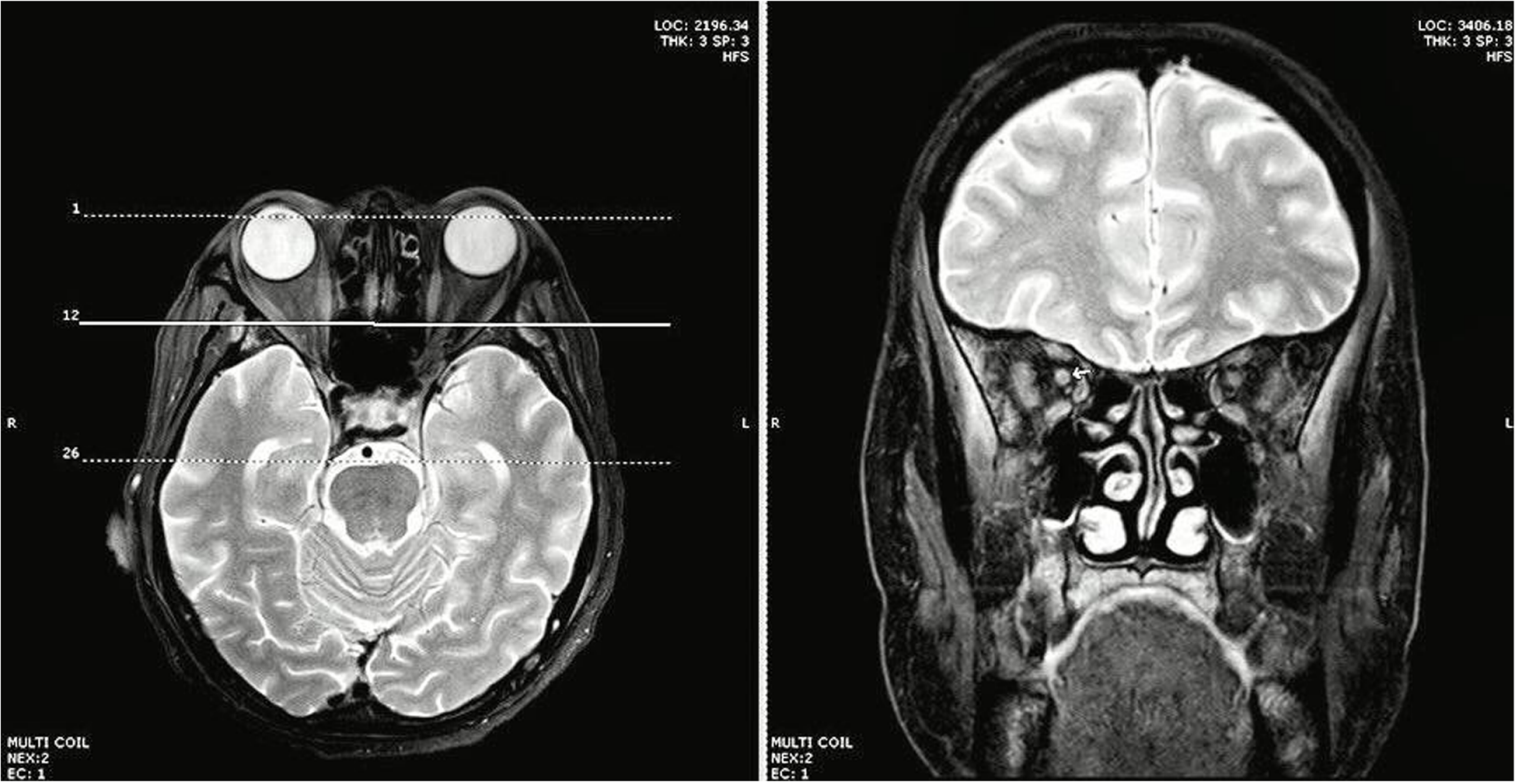
MRI orbits showed relatively small size and peripherally increased signal on T2W/FS at the right optic nerve without definite enhancement

One month later, his eye examination showed bilateral optic disc swelling. Magnetic resonance imaging of orbits was performed and right optic nerve atrophy was identified (Fig. 3). No treatment was required. Two months later, he returned to Siriraj Hospital and complained of vision loss in his left eye. Ophthalmologic examination showed decreased visual acuity in both eyes, and bilateral atypical optic neuritis was suspected. Two days later, he developed a group of painful vesicles at the right popliteal area of S1 dermatome. Lumbar puncture revealed clear cerebrospinal fluid (CSF), a white blood cell count of 1 cell/mm^3^, no RBCs, protein 38 mg/dL, and glucose 59 mg/dL. CSF was sent for VZV-DNA detection by qualitative real-time polymerase chain reaction (PCR) kit (ARGENE®; bioMérieux, Marcy-l'Étoile, France), and the test showed a positive result. Even though the CSF did not present with lymphocytic pleocytosis, a positive CSF analysis for VZV-DNA by PCR is consistent with VZV infection of the central nervous system. In anti-IFN-γ AAbs patients with visual loss, it is indicative of VZV optic neuritis. Moreover, he had a group of vesicles at the right popliteal area. Therefore, he was diagnosed disseminated VZV infection with optic neuritis. He was treated with intravenous acyclovir 1500 mg/day. Two weeks after treatment, his vision and visual acuity were both improved.

## Discussion and conclusions

*Cryptococcus* spp., VZV, and dNTM are intracellular opportunistic microorganisms that most often infect immunocompromised host, especially HIV-infected individuals. However, adult-onset immunodeficiency associated with cell-mediated immune defect should be suspected when these infections are found in patients who are not HIV-infected, and who did not receive immunosuppressive therapy. Adult-onset immunodeficiency due to anti-cytokine autoantibodies associated primarily with infectious manifestations is the most strongly suspected etiology in this patient [1]. Anti-IFN-γ AAbs is a syndrome that is associated with disseminated mycobacterium infection, whereas anti-GM-CSF AAbs is mainly associated with cryptococcosis [3]. As such, requests were sent for both anti-IFN-γ AAbs and anti-GM-CSF AAbs, and the results showed positive only for anti-IFN-γ AAbs. It should be noted that GM-CSF is an important cytokine for inducing terminal differentiation of alveolar macrophages, and also for priming the function of neutrophils against intracellular organisms like *cryptococcus* [3]. In the context of a non-HIV patient with cryptococcosis, it is reasonable to test for AAbs to GM-CSF even though the clinical implication of overlap between anti-IFN-γ and anti-GM-CSF syndromes is currently unknown.

IFN-γ is one of the key cytokines that plays an important role against intracellular pathogens, such as mycobacteria, VZV, and *Cryptococcus*. IFN-γ is secreted by T lymphocytes, including CD4+ and CD8+, and NK cells. IFN-γ can bind to specific receptor leading to activation of Janus kinase (Jak1 and Jak2) followed by phosphorylation and dimerization of STAT1, and then it moves to the nucleus to initiate transcription of IFN-γ and subsequent production of IFN-γ [3]. Genetically inherited disorders of the IFN-γ pathway lead to overwhelming infections by intracellular pathogens, such as *Cryptococcus*, VZV, and nontuberculous mycobacteria, which is similar to the etiology in our case.

Additionally, the majority of patients with auto-IFN-γ AAbs were reported to be Southeast Asians [1, 2, 4–8], which is strongly suggestive of an inherited predisposition. These patients were shown to be strongly associated with HLA-DRB1 and HLA-DQB1 [9], which suggests that the development of AAbs to IFN-γ may be similar to those observed in other HLA-linked autoimmune diseases. Indeed, the mechanism of the production of autoantibodies to IFN-γ remains unclear; however, there are some possible biological explanations. The most possible explanation is that HLA may have an effect on Treg cells during the antigenic presentation process leading to an imbalance in Th1 and Th2, which results in excessive IFN-γ following chronic antigenic stimulation [9]. The upregulation of IFN-γ can then induce reactive B-cells to produce autoantibodies to IFN-γ [10]. If the level of these AAbs is high enough to neutralize IFN-γ, the patient becomes susceptible to overwhelming infection by intracellular pathogens. Nonetheless, genetic association alone is not sufficient to trigger the disease, and it is likely that autoimmunity in individuals carrying susceptible HLA alleles may be influenced by other, as yet unidentified, factors.

Auto-IFN-γ AAbs were reported to be associated with several severe disseminated infections with a prevalence of approximately 90% in Asian adults with opportunistic infections [1]. Skin manifestation is a frequent characteristic of this syndrome, and about 80% of cases develop reactive skin conditions and/or unusual infections of the skin [4]. The characteristics of previously reported cases with anti-IFN-γ syndromes are summarized in Table 1 [6, 7, 11–15]. Here, we report the case of an anti-IFN-γ AAbs patient who presented with unusual cryptococcoma at his left index finger, which was confirmed by skin biopsy and fungal identification. However, reactive skin manifestation, such as neutrophilis dermatosis, was not found in this patient.

**Table 1:** Characteristics of reported patients who had dNTM infection associated with anti-IFN-γ autoantibodies

Author/ Year	Patient	Sex	Age (years)	Ethnicity	Underlying disease	*Mycobacterium* spp.	Co-infection	Organ involvement
Hoflich/2004 [11]	1	F	25	Thai	No	*M. chelonae*	*Burkholderia cocovenenans*	LN, BM, spleen, brain, bone
Kampmann/2005 [12]	2	F	46	English	Crohn’s disease	MAC	No	soft tissue, bone, joint
	3	M	32	South African	No	MAC	No	soft tissue, spine
	4	F	59	English	No	*M. fortuitum*	*Aspergillus*	lung
Patel/2005 [13]	5	F	43	Taiwanese	No	MAC	No	bone, skin, soft tissue
	6	F	45	Filipino	No	MAC, *M. chelonae*	No	LN, lung, skin
	7	F	40	Filipino	Cerebellar venous angioma	*M. abscessus*, *M. fortuitum*, MAC	No	LN, lung
	8	F	66	Filipino	No	*M. abscessus*, MAC	HCV, *Pseudomonas aeruginosa, Enterococcus, Achromobacter*	LN, lung
	9	F	31	Filipino	No	MAC	VZV	appendix, bone, soft tissue, retropharynx
Tanaka/2007 [14]	10	M	54	Japanese	No	MAC	*Streptococcus pyogenes*	LN, bone, lung, stomach, BM, pleura
Koya/ 2009 [15]	11	F	44	Japanese	No	MAC	No	bone, soft tissue
Kampitak/2011 [7]	12	M	56	Thai	Epilepsy	*M. intermedium*	*Penicillium marneffei*	blood, BM, urine, LN, lung, bone, eye
Chetchotisakd/ 2017 [6]	13	M	34	Thai	NA	NA	*Cryptococcus*	lung
	14	F	49	Thai				blood, meninges, skin
	15	F	45	Thai				bone, joint
	16	F	47	Thai				blood
	17	M	32	Thai				bone
	18	F	18	Thai				pleura
	19	F	55	Thai				lung
	20	F	82	Thai				parotid gland
	21	M	34	Thai				bone, joint, lung, skin
	22	F	43	Thai				bone, joint, meninges, skin
Our case	23	M	53	Thai	No	*M. abscessus*	*Cryptococcus*, VZV	skin, CNS, lung, LN, eye

*Abbreviations*: *BM* Bone marrow, *CNS* Central nervous system, *HCV* Hepatitis C virus, *MAC Mycobacterium avium* complex, *LN* Lymph node, *F* Female, *M* Male, *NA* Not available, *VZV* Varicella-zoster virus

In conclusion, this is the first case of triple infection with cryptococcosis, VZV, and dNTM in a patient who tested positive for anti-IFN-γ AAbs and negative for anti-GM-CSF AAbs. This case will increase awareness and heighten suspicion of these infections in patients with the described presentations and clinical characteristics, and this will accelerate diagnosis and treatment.

## Data Availability

All data generated or analyzed during this study are included in this published article.

## Abbreviations

ADA: Adenosine deaminase
anti-GM-CSF AAbs: Anti-granulocyte-macrophage colony-stimulating factor autoantibodies
anti-IFN-γ AAbs: Anti-interferon-gamma autoantibodies
CD: Cluster of differentiation
CSF: Cerebrospinal fluid
CT: Computed tomography
DNA: Deoxyribonucleic acid
dNTM: Disseminated nontuberculous mycobacterial
HIV: Human immunodeficiency virus
HLA: Human leukocyte antigen
IFN-γ: Interferon-gamma
JAK: Janus kinase
RBC: Red blood cell
STAT1: Signal transducer and activator of transcription 1
Th: T helper
VZV: Varicella-zoster virus
WBC: White blood cell
PA: Posteroanterior
RUL: Right upper lobe
MRI: Magnetic resonance imaging
T2W/FS: T2-weighted fat-saturated
RBCs: Red blood cells
